# Annotated computed tomography coronary angiogram images and associated data of normal and diseased arteries

**DOI:** 10.1038/s41597-023-02016-2

**Published:** 2023-03-10

**Authors:** R. Gharleghi, D. Adikari, K. Ellenberger, M. Webster, C. Ellis, A. Sowmya, S. Ooi, S. Beier

**Affiliations:** 1 Faculty of Engineering, University of New South Wales, Kensington, NSW 2052 Australia; 2 Prince of Wales Clinical School of Medicine, UNSW Sydney, Sydney, NSW Australia; 3 Department of Cardiology, Prince of Wales Hospital, Sydney, Australia; 4 Auckland City Hospital, 2 Park Road, Auckland, 1023 New Zealand

**Keywords:** Cardiovascular diseases, Research data, Imaging

## Abstract

Computed Tomography Coronary Angiography (CTCA) is a non-invasive method to evaluate coronary artery anatomy and disease. CTCA is ideal for geometry reconstruction to create virtual models of coronary arteries. To our knowledge there is no public dataset that includes centrelines and segmentation of the full coronary tree. We provide anonymized CTCA images, voxel-wise annotations and associated data in the form of centrelines, calcification scores and meshes of the coronary lumen in 20 normal and 20 diseased cases. Images were obtained along with patient information with informed, written consent as part of the Coronary Atlas. Cases were classified as normal (zero calcium score with no signs of stenosis) or diseased (confirmed coronary artery disease). Manual voxel-wise segmentations by three experts were combined using majority voting to generate the final annotations. Provided data can be used for a variety of research purposes, such as 3D printing patient-specific models, development and validation of segmentation algorithms, education and training of medical personnel and *in-silico* analyses such as testing of medical devices.

## Background & Summary

Coronary artery disease is a leading cause of death worldwide^1^, causing a large body of research to focus on the understanding of coronary anatomy and blood flow, disease progression and treatment options^2–4^. With rapid advancements in computation, additive manufacturing and other technologies capable of taking advantage of virtual organ models, computational models of coronary arteries have been increasingly used in research, including the designing and testing of medical devices, as well as for education and training purposes^5^.

While different modalities can be used to image coronary arteries, only Computed Tomography Coronary Angiography (CTCA) is non-invasive and has sufficient sub-millimetre resolution to allow reconstruction of the small coronary arteries. Therefore it is commonly used and as a result ideal as underlying modality for subsequent image segmentation and virtual coronary artery reconstruction. This commonly required manual refinement after initial automatic threshold due to the small scale, lack of clear contrast with the surrounding tissue and common image artefacts, especially for calcified lesions. Segmentation of the full coronary tree is particularly difficult as even with highest resolution CTCA machines today, distal vessels are only captured via a few image pixels. As a result, despite a wealth of CTCA data available to date, there are extremely few virtual coronary models publicly available and the use of reconstruction workflows on a large-scale patient-specific basis is cost and time intensive.

Traditional segmentation methods are extremely time consuming^6^, generally requiring semi-automated segmentation closely supervised by a human expert to guide the algorithm and correct errors. Additionally, the segmentations produced are highly sensitive to the individual expert and hence consistent segmentation between different experts is difficult. This has led to no public datasets currently available for use in applications that require accurate patient-specific coronary models. Related datasets are limited, including the ‘Visible Heart Project’, which, however focuses on educational images and videos using magnetic resonance imaging. Although access may be provided to limited CTCA images, these are without annotations or reconstructed models^7^. Also available is the ‘The Rotterdam dataset’^8,9^, which is primary public dataset, but focused on stenosis detection and stenosis evaluation with sub-voxel accuracy. This dataset may only be used for its stated purpose of stenosis detection and lumen segmentation, and is also no longer publicly available from the challenge website (https://coronary.bigr.nl/).

To overcome the problems with these traditional segmentation methods, we created high quality segmentations of the coronary arteries, to serve as both a benchmark dataset for newly developed segmentation methods and pre-existing segmentation for further processing, for example investigating differences in helicity between stented idealized and patient-specific vessels^10^. This was part of the ‘Automated Segmentation of Coronary Arteries’ (ASOCA) Challenge^11,12^ we facilitated during the Medical Image Computing and Computer Assisted Intervention (MICCAI) 2020 conference to focus on the development of automated segmentation algorithms using this data, providing a convenient system for submission of results and automated evaluation and ranking.

The coronary artery CTCA images were available to us through the Coronary Atlas^13^, an ongoing collection of CTCA images and associated clinical and demographics data used to investigate differences in coronary anatomy^14^ and haemodynamic behaviour between patients^15–17^. A set of 40 patient-specific coronary artery tree data is provided here, including anonymized CTCA images in .nrdd format, combined high-quality manual voxel annotations derived from 3 experts, and other associated data such as centrelines, smoothed meshes in .stl format and calcification scores. These served as the training dataset for the ASOCA challenge. Our dataset is the only public dataset of annotations and associated data of the full coronary tree in 20 normal and 20 disease cases. Additionally, a separate set of 20 CTCA images (the test set images for the ASOCA challenge) is provided primarily to facilitate participating in the challenge. In order to not compromise the integrity of the challenge, no other information is provided with these images. Researchers can participate on the challenge website (asoca.grand-challenge.org), using the training data to develop segmentation algorithms and submit results to the challenge website for automatic evaluation and scoring.

In summary, the current dataset has several advantages over previously available coronary artery datasets. While our dataset is based solely on CTCA and can not provide sub-voxel segmentation and stenosis identification as accurate as the Rotterdam dataset, we do however provide high quality segmentation of all coronary vessels visible in CTCA. In contrast our dataset is available to all researchers including commercial projects. Further, our inclusion of all arteries larger than 1 mm rather than selected vessel segments allows for expanded applications such as more complex simulations, and more comprehensive training and educational applications. The balanced set of normal and diseased patients ensures effects of disease can be independently studied, as well as ensuring that newly developed segmentation algorithms can robustly handle cases with disease. The dataset is sufficiently large and balanced for training machine learning models. Device manufacturers and researchers with an interest in cardiovascular modelling, prediction and treatment of coronary artery disease can analyse this data directly or combine it with other available datasets. The smooth surface meshes and centrelines can be directly used for computational modelling^16^, directly 3D printed for experiments^18–21^, assist in developing and testing medical devices such as stents^22–24^, and can be used for Virtual Reality applications for education and training^25–27^. Moreover, our dataset allows for the development and benchmarking of new segmentation algorithms aiming to efficiently annotate the coronary arteries automatically as per ASOCA challenge^28^.

## Methods

### Patient cohort

Forty patients were randomly selected from a retrospective dataset based on the calcification, stenosis and image quality reported by the cardiologist. Images must have acceptable quality as described by the cardiologist. The dataset was divided into twenty normal patients with no evidence of stenosis and non-obstructive disease, and twenty patients with evidence of calcium scores higher than 0 and obstructive disease. The calcification score in the diseased group ranged between 1 and 986 with a mean of 254. Obstructions in the diseased group ranged from 30% to 70% stenosis. Patients were included during routine procedures after written and informed consent and approval from University of New South Wales Human Research Ethics Committee (Ref. 022961).

### Imaging

The CTCA imaging was undertaken using a multi-detector CT scanner (GE Lightspeed 64 multi-slice scanner, USA) using retrospective ECG gating. A contrast medium (Omnipaque 350) was used for imaging and the patient heart rate was controlled around 60bpm by administration of beta blockers. The end diastolic time step was saved for analysis and the images exported as DICOM files. Images were converted to Nearly Raw Raster Data (NRRD) format during the anonymization process and the intensity rescaled to Hounsfield units based on the appropriate DICOM tags.

### Annotation

**Fig. 1:**
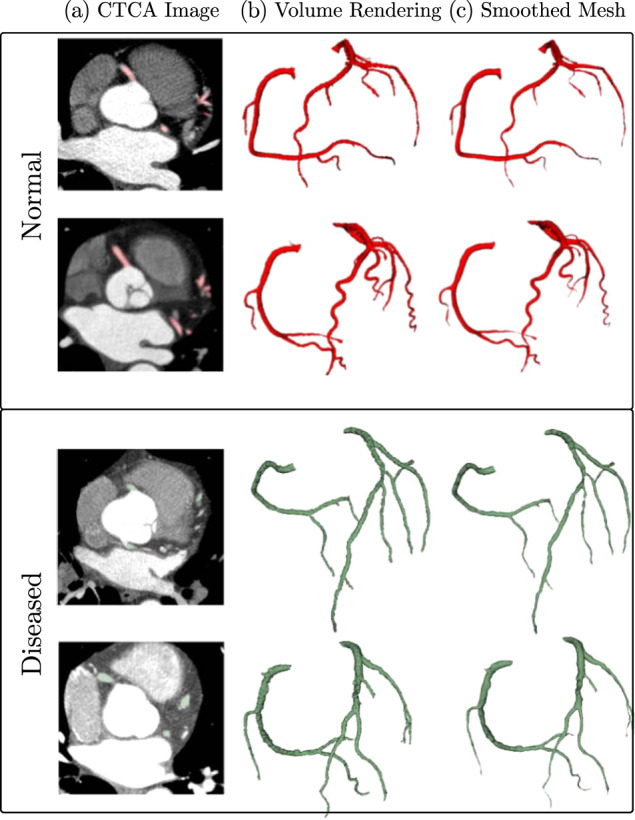
Samples of annotated data showing (**a**) an annotated slice of the CTCA images, (**b**) volumetric rendering of the labelled voxels, and (**c**) smooth surface mesh (left to right) generated from the normal (top two rows) and diseased (bottom two rows) coronary artery image annotations.

**Fig. 2:**
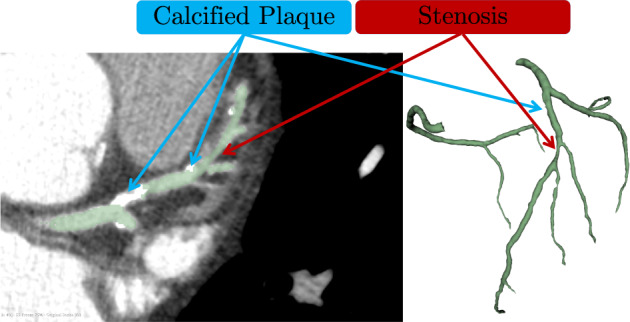
Calcified and non-calcified plaques present in the dataset.

The open-source software 3D Slicer (version 4.3)^29^ was used to manually annotate the coronary arteries images. The annotation process was performed independently by three annotators, who were instructed to segment the left and right coronary trees starting at the aortic root. Thresholding at a cut-off chosen by the expert was used to generate an initial rough segmentation of the vessel, followed by manually correcting the vessel contours in each slice. All coronary vessels with a diameter larger 1 mm, representing 1–2 voxels, were included in the segmentation. In segments showing significant imaging artefacts affecting the vessel that would make further segmentation unfeasible, the rest of the vessel was ignored. A sample of the annotated CTCA images and the resulting 3D reconstructions are shown in Fig. 1. Figure 2 shows a diseased case with calcified plaque and stenosis.

### Post processing

**Fig. 3:**
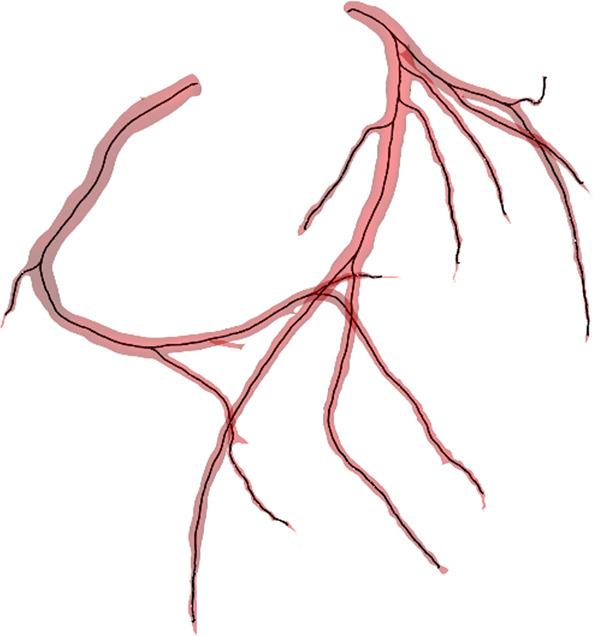
Sample coronary tree surface and centreline.

The annotations are combined to produce a final segmentation of the arteries by majority vote among the three annotations, i.e. including regions where at least two of the annotators agreed. Small vessels (<1 mm, i.e. 1–2 voxels) were removed if they were mistakenly included. The segmentations are available as voxel-wise annotations, as well as smoothed surface meshes. Surface meshes were produced from the annotations using the Flying Edges algorithm^30^. It should be noted that with voxel-wise labelling as used in this dataset rather than a tubular parametrization, further smoothing would be necessary to recover a smooth vessel shape. The annotations were smoothed using Taubin’s algorithm^31^, implemented in the open-source Vascular Modelling Tool Kit (VMTK, https://www.vmtk.org)^32,33^, with a passband of 0.03 and 30 iterations before being exported as an STL file. Taubin’s smoothing method is commonly used when processing vessel segmentations^34^ and is expected to preserve topology and volume of the vessels^35^. These settings correspond to the smoothing used in the Coronary Atlas to calculate shape parameters. The raw annotations provided can be used to produce surface meshes with different smoothing settings if needed. Vessel centrelines were extracted manually by marking the inlet and outlet points on the mesh for automated centreline calculation in VMTK, as shown in Fig. 3.

### ASOCA test data set

An additional 10 normal and 10 diseased CTCA cases, separate to the 20 normal and 20 diseased used for the training data, were selected based on the same criteria to serve as the test set for the ASOCA challenge. These cases will be distributed alongside the annotated dataset to facilitate further participation in the challenge. Ground truth annotation and other associated data for these cases is not publicly available.

## Data Records

The dataset is available on the UK Data Service (https://reshare.ukdataservice.ac.uk/855916/)^36^. Patients are labelled sequentially from 1 to 20, with normal and diseased patients labelled separately (i.e. Normal_1…Normal_20 represent the normal patients and Diseased_1…Diseased_20 represent diseased patients). CTCA scans are provided as Nearly Raw Raster Data (NRRD) file labelled sequentially based on patient name (Normal_1.nrrd, Normal_2.nrrd…). This naming convention is used for the rest of the data folders. The annotations folder contains the final annotation for each patient. This represents the voxel-wise annotations, with the background voxels assigned a value of 0 and the foreground (vessel lumen) assigned a value of 1. Both the CTCA images and annotations have anisotropic resolution, a common characteristic of most CT machines, with the z-axis resolution of 0.625 mm and the in-plane resolution ranging from 0.3 mm to 0.4 mm depending on the patient. The SurfaceMeshes directory contains smooth surface meshes generated from the voxel annotations. These meshes are provided in STL format, with an average of 37,000 vertices to capture the arterial geometry.

The centrelines folder contains centrelines of the coronary arteries for each patient, provided in VTK Poly Data (VTP) format that allows for efficient storage of centreline data. Figure 3 shows a sample of the extracted centreline and underlying surface mesh. The spreadsheet DiseaseReports.xlsx reports calcium score and stenoses levels for each patient.

## Technical Validation

Dice Similarity Coefficient (DSC)^37^ is frequently used to measure the degree of overlap between annotations. DSC is defined as in Eq. 1 for two sets of voxels A and B. Similarly, Hausdorff Distance (HD) as shown in Eq. 2 measures the distance of corresponding points between annotations. In practice commonly the 95th percentile HD is used rather than the maximum in order to reduce sensitivity to outliers^38^.

1 $${\rm{DSC}}=\frac{2| A\cap B}{| A| +| B| }$$

2 $${\rm{HD}}={\rm{\max }}(\mathop{{\rm{\max }}}\limits_{x\in A}\,\mathop{{\rm{\min }}}\limits_{y\in B}d(x,y),\mathop{{\rm{\max }}}\limits_{y\in A}\,\mathop{{\rm{\min }}}\limits_{x\in B}d(x,y))$$

We used DSC (Table 1) and 95th percentile HD (Table 2) to compare variability between annotators compared to the final ground truth generated for each case. The average Dice Score among the three annotators was 85.6% ± 7.7% (mean ± standard deviation) and an average HD of 5.92 ± 7.3 mm (mean ± standard deviation). The concordance between annotators was higher for normal cases compared to diseased (87.4% vs 83.9%, p = 0.01 using Welch’s t test^39^), due presence of stenosis and calcified plaques complicating the annotation of diseased images. Hausdorff Distance showed similar results (4.45 mm in normal cases vs 7.38 mm in diseased, p = 0.028). A Dice Score of 1 (indicating perfect agreement) is difficult to achieve, as this dataset attempts to segment the full coronary artery tree including small arteries near the limit of CTCA imaging resolution. This Dice Score and Hausdorff Distance indicates high agreement between the annotators and is unlikely to adversely affect usage of this dataset. Table 3 shows the Hausdorff Distance between centre of the voxel labels and the smoothed mesh.

**Table 1:** Annotator Dice Similarity Coefficient for each patient.

Normal				Diseased			
Patient	Annotator 1 (%)	Annotator 2 (%)	Annotator 3 (%)	Patient	Annotator 1 (%)	Annotator 2 (%)	Annotator 3 (%)
#1	95.1	91.8	92.3	#1	84.2	82.9	86.0
#2	79.3	82.4	93.0	#2	84.7	81.1	86.6
#3	96.7	85.9	77.3	#3	71.8	83.6	86.0
#4	96.7	75.6	81.3	#4	92.7	89.8	67.7
#5	86.1	90.4	93.4	#5	96.3	87.7	83.1
#6	91.7	81.2	97.4	#6	83.9	83.8	87.2
#7	91.6	86.4	93.6	#7	84.9	76.3	91.3
#8	87.8	82.4	90.5	#8	86.4	79.4	83.4
#9	97.7	73.5	84.9	#9	90.3	82.0	84.0
#10	95.7	89.8	95.0	#10	82.2	79.1	84.0
#11	88.3	93.5	86.1	#11	84.2	80.5	85.3
#12	92.4	78.9	87.6	#12	80.4	82.2	85.9
#13	98.1	92.8	70.4	#13	83.1	87.6	79.6
#14	96.2	90.2	57.2	#14	88.3	89.7	81.4
#15	98.0	77.9	67.3	#15	76.4	85.7	85.1
#16	98.4	72.8	93.8	#16	79.4	76.9	89.4
#17	97.0	78.4	85.3	#17	90.3	88.7	71.8
#18	91.9	92.8	60.2	#18	78.1	90.6	88.1
#19	87.2	86.2	92.7	#19	80.8	85.0	85.4
#20	90.5	92.2	97.4	#20	82.0	87.3	83.3

**Table 2:** Annotator 95^th^ percentile Hausdorff Distance for each patient.

Normal				Diseased			
Patient	Annotator 1 [mm]	Annotator 2 [mm]	Annotator 3 [mm]	Patient	Annotator 1 [mm]	Annotator 2 [mm]	Annotator 3 [mm]
#1	0.42	4.07	0.42	#1	9.2	5.12	2.3
#2	1.1	4.86	0.62	#2	4.0	14.36	0.44
#3	0.0	3.56	5.58	#3	11.91	3.61	0.62
#4	0.0	5.74	0.62	#4	0.62	10.29	7.29
#5	0.72	3.42	8.16	#5	0.45	11.31	5.55
#6	2.17	7.82	0.0	#6	0.62	10.08	1.4
#7	6.92	1.31	0.4	#7	0.56	12.12	0.56
#8	0.57	1.49	4.78	#8	37.46	15.92	14.56
#9	0.0	21.78	0.97	#9	7.76	5.56	2.82
#10	0.44	10.79	8.11	#10	0.73	3.32	11.66
#11	2.92	1.4	1.78	#11	0.7	12.11	1.68
#12	0.73	12.61	0.52	#12	2.34	6.01	0.7
#13	0.0	0.33	2.35	#13	41.0	3.39	1.53
#14	0.37	2.4	13.87	#14	14.37	8.47	1.21
#15	0.0	9.98	10.51	#15	1.8	6.74	0.9
#16	0.0	16.87	0.35	#16	29.7	15.0	0.62
#17	0.36	6.14	21.86	#17	0.39	5.3	15.92
#18	10.86	3.57	19.62	#18	21.26	2.6	0.65
#19	3.15	13.76	0.38	#19	5.13	13.0	3.16
#20	0.42	3.29	0.0	#20	4.87	6.87	3.64

**Table 3:** 95^*th*^ percentile Hausdorff Distance between smoothed meshes and voxel labelmap.

Patient #	#1	#2	#3	#4	#5	#6	#7	#8	#9	#10	#11	#12	#13	#14	#15	#16	#17	#18	#19	#20
Normal [mm]	1.39	1.13	1.40	1.05	1.16	1.45	1.35	1.40	1.24	1.49	1.28	1.24	1.31	1.24	1.40	1.38	1.22	1.25	1.13	1.20
Diseased [mm]	1.10	1.03	1.15	1.40	1.36	1.14	1.19	1.06	1.30	1.08	1.17	1.17	1.29	1.28	1.05	1.43	1.05	1.51	1.31	1.34

## Usage Notes

These recommendations focus on free, open-source software, however as the dataset is provided commonly used formats commercially available software suites will can also be utilised. CTCA and ground-truth data is provided in NRRD format, compatible with all common medical imaging software such as 3D Slicer^29^ and ITK-SNAP^40^. 3D Slicer is the recommended software for working with this data, providing tools for common editing operations and various add-ons for specialised tasks. The centrelines are saved in VTK Poly Data (VTP) format, expected to be used with the Visualization Toolkit (VTK)^41^ and the Vascular Modelling Toolkit^32,33^. VMTK is also available as a 3D Slicer add-on. Surface meshes are provided in Standard Tessellation Language (STL), compatible with most mesh software. Both 3D Slicer and VMTK allow editing and processing STL meshes, including addition of flow extensions and generation of volume meshes for computational fluid dynamics simulations. Specific mesh editing software such as Meshlab^42^ can be used for more complex tasks. The dataset can be also be used to develop new segmentation algorithms and evaluate the performance on the standardised ASOCA challenge. Submission instructions are available on the challenge website (https://asoca.grand-challenge.org/SubmittingResults/).

The dataset can be used for research and commercial purposes. Researchers request access on the UK Data Service^36^ for access and provide evidence of ethics review and approval, or waiver regarding their project.

## Data Availability

The code for creation of this dataset, usage examples and evaluation code used in the challenge is available on GitHub (https://github.com/Ramtingh/ASOCADataDescription). Figures 1–3 were created with data included in the dataset. A copy of the raw data used is included in the repository under the corresponding folder to maker recreating these figures easier. 3D Slicer (version 4.3) was used in the preparation of the dataset and Figs. 1 and 2. Vascular Modelling Tool Kit (version 1.4) was used to calculate centerlines and generate Fig. 3.
